# A Rare Case of Pulmonary Ossification Findings That Rapidly Progressed and Improved During the Course of COVID‐19 Pneumonia

**DOI:** 10.1002/rcr2.70582

**Published:** 2026-04-12

**Authors:** Chie Hirama, Arisa Mitsuo, Yuichi Kato, Iori Motohashi, Kenya Ie, Taku Tanaka, Chiaki Okuse, Shigeki Fujitani

**Affiliations:** ^1^ Medical Emergency and Disaster Center Kawasaki Municipal Tama Hospital Kawasaki Kanagawa Japan; ^2^ Center for General Medicine Kawasaki Municipal Tama Hospital Kawasaki Kanagawa Japan; ^3^ St. Marianna University School of Medicine Kawasaki Kanagawa Japan

**Keywords:** COVID‐19, pneumonia, pulmonary ossification

## Abstract

An 88‐year‐old man with a history of myocardial infarction, non‐valvular atrial fibrillation and hypothyroidism was admitted to our hospital with a diagnosis of coronavirus disease. On admission, he had hypoxemia, with peripheral blood oxygen saturation at 95% on 5 L of oxygen via face mask ventilation. Chest computed tomography (CT) showed bronchial wall thickening and emphysema in both lungs, ground‐glass opacities and high‐attenuation areas in the bilateral lower lungs. After hospitalization and treatment, hypoxemia improved; however, chest CT showed marked progression of the high‐attenuation areas, suggestive of pulmonary ossification (PO) in both lower lungs. The patient was transferred to a rehabilitation hospital; however, hypoxemia had improved at the time of discharge and chest CT showed marked improvement in the findings suggestive of PO.

## Graphical abstract

We report a case of pulmonary ossification findings that rapidly progressed and improved during the course of COVID‐19 pneumonia.

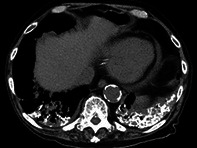

## 1 Introduction

To our knowledge, there are few reports of pulmonary calcification in coronavirus disease (COVID‐19) pneumonia, especially pulmonary ossification (PO) [1]. PO is often asymptomatic or progresses slowly. We report a case of PO findings that rapidly progressed and improved during the course of COVID‐19 pneumonia on chest computed tomography (CT).

## 2 Case Report

An 88‐year‐old man with a history of myocardial infarction, non‐valvular atrial fibrillation and hypothyroidism presented at the clinic with a one‐week history of fever. Chest CT performed 5 days before admission showed only slight ground‐glass opacities (GGO) in the right lower lobe (Figure 2a). Two days before admission, a COVID‐19 polymerase chain reaction test was positive. He was referred to our hospital for COVID‐19 and admitted due to hypoxemia requiring 5 L oxygen administration. Wheezing was heard on auscultation of both lungs.

**FIGURE 1:**
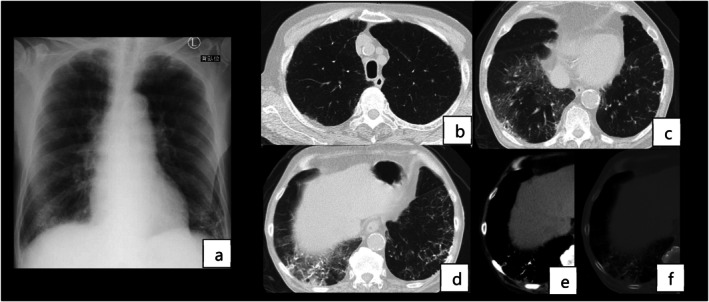
Chest radiography and computed tomography images on admission.

The laboratory findings are shown in Table 1. Chest radiography showed bilateral reticular and granular shadows in the lower lungs (Figure 1a). Chest CT showed emphysematous changes, GGO in the right middle and lower lobes and fine granular and branched high‐attenuation areas in the lower lungs, which were also identifiable on bone window images, suggestive of PO (Figure 1b–f).

**TABLE 1:** Laboratory findings.

Cell blood count			Biochemistry			Immunology		
WBC	10.2	×10^3^/μL	T‐Bil	1.1	mg/dL	BNP	463.4	pg/mL
neutrophills	77.5	%	AST	13	U/L	Ferritin	540	ng/mL
lymphocytes	10.9	%	ALT	6	U/L	Procalcitonin	0.04	ng/mL
monocytes	9.2	%	LDH	260	U/L	KL‐6	310	U/mL
eosinophills	2.1	%	ALP	191	U/L	SP‐D	190	ng/mL
basophills	0.3	%	γGTP	14	U/L	RF	< 5	IU/mL
Hb	12.4	g/dL	CK	36	IU/L	IgG	1033	mg/dL
Hct	37.3	%	TP	6.4	g/dL	IgA	320	mg/dL
Plt	27.6	×10^4^/μL	ALB	2.6	g/dL	IgM	42	mg/dL
Coagulation			BUN	40.8	mg/dL	IgE	< 5	U/L
PTINR	1.5		Cr	1.34	mg/dL	C3	130	IU/mL
APTT	35.1	s	UA	6.3	mg/dL	C4	39	mg/dL
Fib	1000	mg/dL	Na	139	mEq/L	ANA	< 40	
D‐dimer	0.6	μg/mL	Cl	103	mEq/L	Anti SS‐A antibody	< 1.0	U/mL
Venous gas analysis (room air)			K	4.6	mEq/L	Anti ARS antibody	< 5.0	U/mL
pH	7.42		Ca	8.1	mg/dL	Anti CCP antibody	< 0.5	U/mL
PaCO_2_	41.9	Torr	P	3.4	mg/dL	MPO‐ANCA	< 0.5	U/mL
HCO3−	26.5	mmol/L	Mg	2.7	mg/dL	PR3‐ANCA	< 0.5	U/mL
Lac	1.2	mmol/L	Glucose	134	mg/dL	BNP	463.4	pg/mL
			CRP	16.09	mg/dL	Ferritin	540	ng/mL
			SAA	1452.5	μg/mL	Procalcitonin	0.04	ng/mL
			ACE	8.5	IU/L	KL‐6	310	U/mL

Abbreviations: ACE, Angiotensin I converting enzyme; ANA, antinuclear antibody; ARS, aminoacyl tRNA synthetase; CCP, cyclic citrullinated peptide; CRP, C reactive protein; MPO‐ANCA, myeloperoxidase anti‐neutrophil cytoplasmic antibody; PR3‐ANCA, proteinase3 anti‐neutrophil cytoplasmic antibody; RF, rheumatoid factor; SAA, serum amyloid A protein.

**FIGURE 2:**
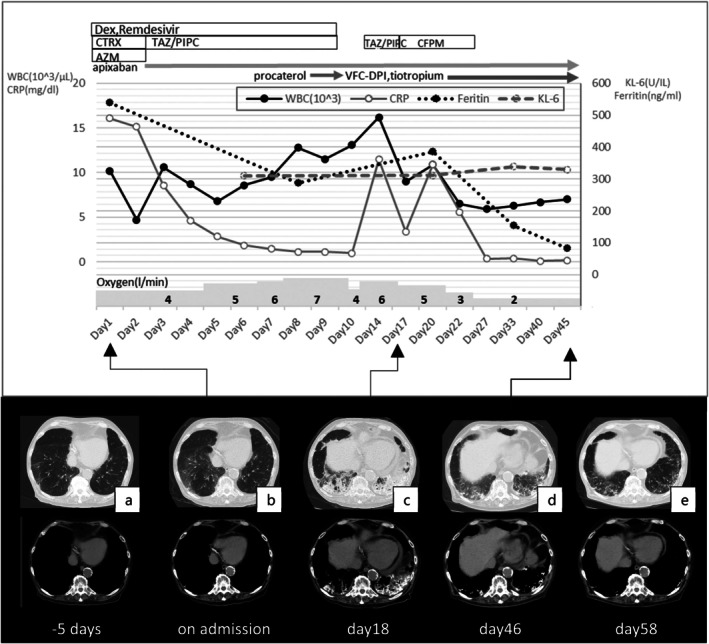
Clinical course and changes of imaging findings.

Remdesivir and dexamethasone were administered for 10 days. Ceftriaxone, azithromycin and tazobactam piperacillin (T/P) were also administered for bacterial pneumonia. The patient's respiratory status deteriorated, and he required high‐flow oxygen on Day 7. Chest CT on Day 8 showed fibrotic changes and a slightly worsened high‐attenuation area, suggestive of PO. Since the patient had wheezing, various inhaled therapies were added for hypoxemia (Figure 2).

On Day 14, CRP remained elevated and chest X‐ray showed a new shadow in the right lower lung, so T/P was resumed for nosocomial pneumonia.

By Day 18, the hypoxemia had improved to 4 L of oxygen and chest CT showed improved fibrotic changes; however, volume loss in the lower lobes and high‐attenuation area had markedly worsened (Figure 2c). On Day 46, when the patient was transferred to a rehabilitation hospital, hypoxemia had improved to 2 L of oxygen and chest CT showed an improved high‐attenuation area, although volume loss persisted (Figure 2d).

On Day 58, the patient was weaned off oxygen and discharged to a nursing home. The high‐attenuation area on chest CT had markedly improved to the same level as at the time of admission (Figure 2e).

## 3 Discussion

Herein, we report a case of PO findings that rapidly progressed and improved during the course of COVID‐19 pneumonia.

PO manifests as the presence of mature bone in the lungs, often including the bone marrow, and can be seen under bone window images (WW 2000 HU, WL 500 HU) of the chest CT. PO can be nodular or dendriform [2].

Intrapulmonary calcification is usually seen in the upper lobes and is depicted as centrilobular‐GGO or multiple calcium nodules by chest CT. PO is seen in the lower lobes, especially in dendritic PO; metastatic calcification is not detectable in bone window images. PO can also be distinguished by its different distribution and linear lesions from dystrophic calcification.

The distribution of the lesions, which are primarily located just beneath the pleura in the basal portion of the lower lobe of the lungs and in the alveolar interstitium and septum, resembles that of the chest CT findings on admission for out patients. PO is typically asymptomatic or slowly progressing; therefore, it is rarely diagnosed before death.

A case of PO caused by alveolar haemorrhage that improves with steroid therapy has been reported [3]. This case is rare in that the PO progressed rapidly and the lesions disappeared during the course, and volume loss improved.

Takeda et al. reported a case of PO seen as a high‐attenuation area caused by atelectasis and further reported better visibility of the PO using chest CT due to lung volume loss [4].

Some researchers speculated that the mechanism of PO signifies the transformation of lung fibroblasts and macrophages into osteoblasts and osteoclasts due to hypoxia and decreased lung compliance as a consequence of lung injury [2].

TGF‐β, a growth factor produced by inflammatory macrophages and damaged epithelial cells, is involved in the production and deposition of extracellular matrix and fibrosis of the lung and other tissues, and promotes the proliferation of osteoblasts and chondroblasts.

COVID‐19 can cause lung damage, which is characterized by diffuse alveolar damage resembling that in acute respiratory distress syndrome, release of various cytokines including TGF‐β and initiation of tissue fibrosis [5], which leads to the formation and proliferation of osteoblasts and chondroblasts, resulting in PO.

However, the fact that ossification, fibrosis, and exacerbation of the lesions were caused by repeated lung injury, with no changes observed in KL‐6 levels during the disease and or chest CT findings after the transfer, suggests that atelectasis may have allowed visualization of the PO and exacerbation followed by improvement of the lesions. The most plausible explanation is that CT findings of PO became apparent due to atelectasis, suggesting that respiratory function may recover even with a rapid deterioration of lung damage and progression of COVID‐19 pneumonia.

The limitations of this report include that a chest CT scan was not performed before the onset of COVID‐19 and that a lung biopsy could not be performed due to the patient's worsening respiratory condition.

PO often has a chronic course and is asymptomatic and undiagnosed, but sometimes appears to deteriorate rapidly due to atelectasis. Therefore, patients with PO should continue treatment with the expectation of improved respiratory function.

## Author Contributions

All authors have read and approved the final version of the manuscript.

## Consent

The authors declare that written informed consent was obtained for the publication of this manuscript and accompanying images using the form provided by the Journal.

## Conflicts of Interest

The authors declare no conflicts of interest.

## Data Availability

The data that support the findings of this study are available from the corresponding author upon reasonable request.
